# Ferroptosis nanomedicine for cancer therapy: strategies and challenges

**DOI:** 10.3389/fmolb.2026.1950781

**Published:** 2026-09-23

**Authors:** Yanpin Ma, Ziyi Xu, Hudabia Akram, Tiancheng Yang, Jiachun Sun, Xinyu Gu

**Affiliations:** Henan Key Laboratory of Cancer Epigenetics, Cancer Institute, The First Affiliated Hospital, College of Clinical Medicine, Henan University of Science and Technology, Luoyang, Henan, China

**Keywords:** cancer therapy, ferroptosis, immunotherapy, nanomedicine, tumor microenvironment

## Abstract

Ferroptosis is an iron-dependent form of regulated cell death driven by lipid peroxidation, and it has become an attractive strategy for cancer therapy because it can target redox and metabolic vulnerabilities that differ from those exploited by apoptosis-based treatments. Small-molecule ferroptosis inducers have clarified the therapeutic logic of this pathway, but their use in cancer treatment requires control over pharmacokinetics, tumor selectivity, local activation and systemic safety. Nanomedicine offers a programmable route to address these requirements. Engineered nanoplatforms can supply catalytic iron, remodel the tumor redox state, deplete antioxidant defenses, carry ferroptosis inducers, respond to tumor microenvironmental cues and combine ferroptosis with phototherapy, sonodynamic therapy, chemotherapy, radiotherapy or immunotherapy. Yet the field remains largely preclinical, and translation will depend on biodistribution, degradability, metal-related toxicity, manufacturing reproducibility, pharmacodynamic biomarkers and rational patient selection. This review summarizes the biological rationale for ferroptosis-targeted cancer therapy, analyzes major translational barriers, and synthesizes nanomedicine strategies that may move ferroptosis induction from experimental proof of concept toward clinically interpretable intervention.

## 1 Introduction

Cancer therapy increasingly depends on exploiting regulated cell-death programs that are not fully captured by classical apoptosis-centered models. Necroptosis, pyroptosis, ferroptosis and cuproptosis have expanded the therapeutic vocabulary of oncology by linking cell death to inflammatory signaling, metal metabolism, redox balance and immune remodeling (Tong et al., 2022). Beyond ferroptosis, nanomedicine-enabled pyroptosis has also emerged as a promising immunogenic cell death modality for cancer immunotherapy, with recent reviews comprehensively covering material design strategies and synergistic mechanisms (Wei et al., 2026). Ferroptosis is particularly relevant because malignant cells often alter iron handling, lipid metabolism and antioxidant capacity during proliferation, metastatic adaptation and treatment resistance (Bae et al., 2026; Wang et al., 2022). Its core biochemical signature, iron-dependent lipid-peroxide accumulation, offers a mechanistic route for killing tumor states that resist conventional cytotoxic stress (Nie et al., 2022).

Nanomedicine has become a major platform for translating ferroptosis biology into cancer therapy because it can coordinate cargo loading, tissue distribution, local activation and multimodal treatment in one design (Ikram and Rasheed, 2023). Reviews of ferroptosis-directed nanoparticles have emphasized that nanocarriers can improve solubility, protect labile inducers, enrich drugs in tumors and integrate diagnostic or therapeutic functions (Zaffaroni and Beretta, 2021). Self-assembled ferroptosis nanomedicines extend this logic by reducing carrier burden and by building therapeutic activity into the supramolecular structure itself (Ma et al., 2023). Broader summaries of nanomaterial-based ferroptosis therapy also show that the field has moved from simple drug delivery toward redox engineering, catalytic therapy and combination regimens (Liu et al., 2023).

Tumor ferroptosis depends on iron availability, polyunsaturated lipid peroxidation, glutathione peroxidase 4 (GPX4) activity, glutathione (GSH) metabolism, parallel defense pathways and tumor microenvironment (TME) conditions (Lee and Roh, 2025; Yang F. et al., 2024). Nanomedicine-mediated ferroptosis strategies therefore differ from conventional single-agent approaches because they can alter several of these variables at once (Yin et al., 2023). In lung cancer and other solid tumors, recent reviews have framed this approach as an opportunity to combine ferroptosis induction with imaging, immune activation, phototherapy and chemotherapy rather than as a standalone cytotoxic mechanism (Xu Y. et al., 2025).

This review provides a translation-oriented synthesis of ferroptosis-targeted cancer nanomedicine. It first summarizes the biological rationale for targeting ferroptosis and the delivery, toxicity and manufacturing barriers that constrain ferroptosis inducers. It then integrates nanoplatform designs that directly induce ferroptosis, enhance precision delivery, respond to the TME and enable synergistic therapy. Finally, it discusses biosafety, pharmacokinetic standardization, biomarkers and design principles needed for clinically interpretable ferroptosis nanomedicine.

## 2 Biological rationale and translational barriers

Ferroptosis-targeted cancer therapy rests on a clear biological premise but faces a practical translational problem. The pathway is mechanistically tractable because iron metabolism, lipid peroxidation and antioxidant defenses can be perturbed by defined molecular or material interventions. The same pathway is difficult to translate because systemic lipid peroxidation, heterogeneous tumor accumulation and complex bio-nano interactions can weaken efficacy or create toxicity (Figure 1).

**FIGURE 1:**
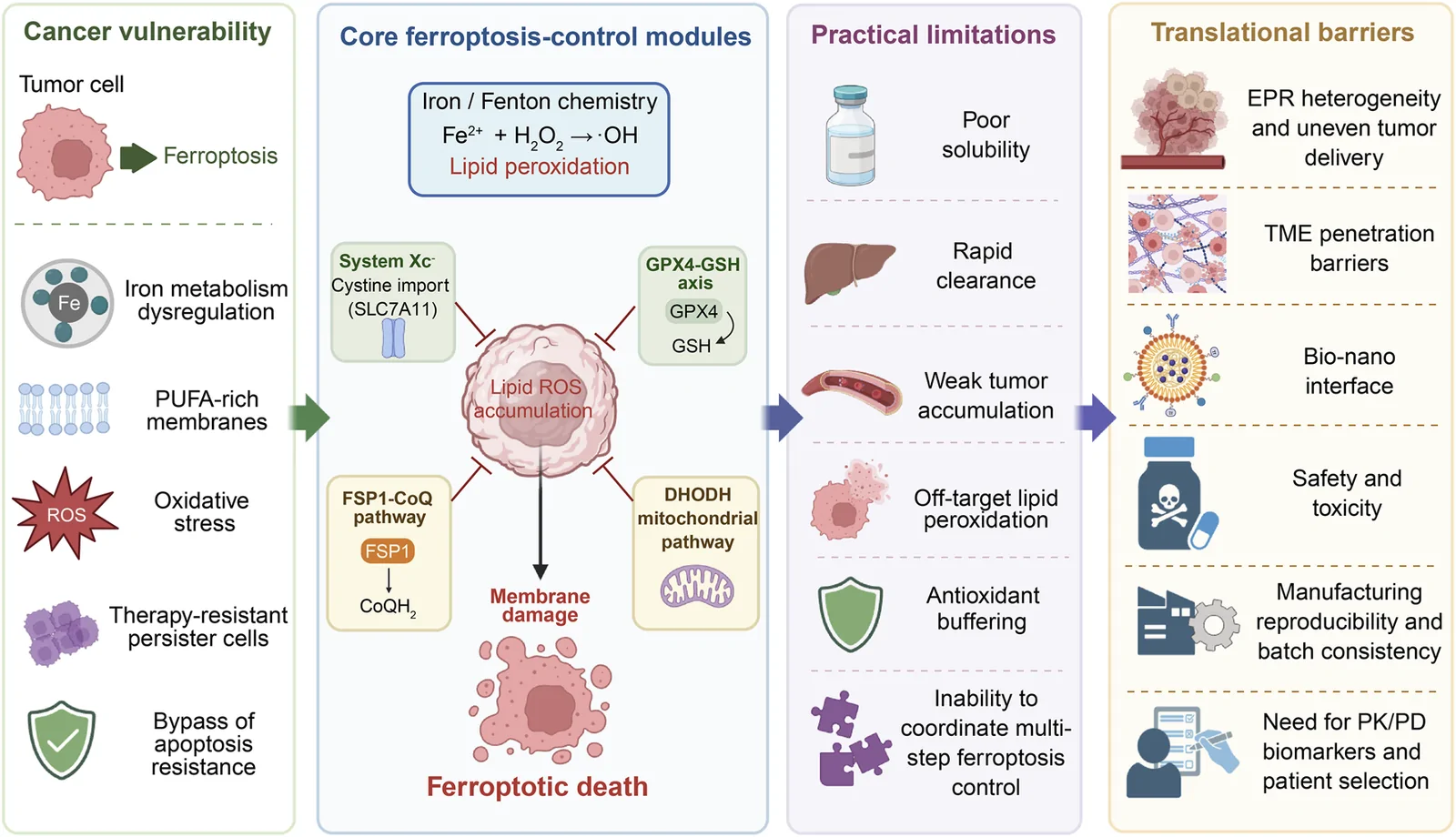
Biological rationale and translational barriers of ferroptosis-targeted cancer therapy. The figure summarizes iron metabolism, lipid peroxidation, GPX4-GSH, FSP1-CoQ and DHODH defense pathway, and links them to drug delivery, toxicity, EPR heterogeneity, TME penetration and manufacturing barriers. Abbreviations: CoQH_2_, reduced coenzyme Q10 (ubiquinol); DHODH, dihydroorotate dehydrogenase; EPR, enhanced permeability and retention; FSP1, ferroptosis suppressor protein 1; GPX4, glutathione peroxidase 4; GSH, glutathione; PUFA, polyunsaturated fatty acid; TME, tumor microenvironment.

### 2.1 Ferroptosis as a cancer vulnerability

Ferroptosis was defined as an iron-dependent form of nonapoptotic cell death with biochemical and morphological features distinct from apoptosis, establishing a new framework for targeting redox fragility in diseased cells (Dixon et al., 2012; Karaseva et al., 2024). The pathway connects metabolism, iron handling and membrane lipid injury, which explains why it is relevant to cancer biology rather than a narrow chemical phenotype (Stockwell et al., 2017). Broad reviews of regulated cell death in cancer place ferroptosis among several nonapoptotic programs that may bypass resistance to apoptosis-directed therapies.

The therapeutic value of ferroptosis in cancer is strengthened by evidence that it intersects with tumor suppression and drug tolerance. p53-mediated tumor-suppressive activity can involve ferroptosis-related control of redox and cystine metabolism, linking ferroptosis to canonical cancer biology (Jiang et al., 2015). Drug-tolerant persister cancer cells show vulnerability to GPX4 inhibition, indicating that ferroptosis can expose survival states generated by therapy pressure (Hangauer et al., 2017). Imidazole ketone erastin improved the pharmacological behavior of an erastin-like inducer and slowed tumor growth in a mouse lymphoma model, showing that inducer optimization can move ferroptosis chemistry toward *in vivo* therapy (Zhang et al., 2019).

### 2.2 Core ferroptosis-control pathway

Ferroptosis sensitivity is controlled by a balance between lipid-peroxide production and antioxidant defense. GPX4 suppresses ferroptotic cancer-cell death by reducing lipid hydroperoxides, making GPX4 a central checkpoint for ferroptosis induction (Yang et al., 2014). Ferroptosis suppressor protein 1 (FSP1) acts as a glutathione-independent suppressor pathway, adding a parallel defense layer beyond the canonical GPX4-GSH system (Doll et al., 2019). The FSP1-CoQ oxidoreductase pathway can inhibit ferroptosis in parallel with GPX4, which implies that effective ferroptosis therapies may require simultaneous disruption of redundant defense systems (Bersuker et al., 2019). Dihydroorotate dehydrogenase (DHODH) provides another ferroptosis-defense mechanism and represents a targetable vulnerability in cancer, particularly in contexts where mitochondrial lipid-peroxide control becomes limiting (Mao et al., 2021).

These can explain why ferroptosis nanomedicine often combines iron delivery, GSH depletion, GPX4 suppression and redox catalysis. A nanoplatform that only supplies iron may be neutralized by high antioxidant capacity, whereas a platform that only inhibits GPX4 may fail when iron availability or lipid-peroxide propagation is insufficient. The most persuasive nanomedicine designs therefore treat ferroptosis as a networked death program rather than as a single molecular switch (Figure 1).

### 2.3 Drug delivery and bio-nano barriers

The promise of ferroptosis induction is constrained by drug delivery. Cancer nanomedicine has made progress in improving drug distribution and tumor exposure, but clinical translation is shaped by formulation complexity, biological heterogeneity, immune recognition and manufacturing control (Shi et al., 2017). The enhanced permeability and retention (EPR) effect can support tumor accumulation, yet it is variable across tumors and patients, so passive targeting alone is insufficient as a universal design principle (Shi et al., 2020). Endothelial permeability contributes to EPR behavior, but vessel phenotype and TME structure determine whether nanoscale carriers can reach therapeutically relevant tumor regions (Lahooti et al., 2023).

Ferroptosis nanomedicine adds an additional safety problem because the intended mechanism, lipid peroxidation, is not tumor-exclusive. Ferroptosis nanomedicine reviews have identified clinical challenges related to metabolic remodeling, immune effects, safety and the need for better translational criteria (Yang et al., 2023). Studies of chemotherapy-associated acute kidney injury show that ferroptosis can contribute to normal-organ injury, which supports careful avoidance of systemic ferroptosis activation (Li J. et al., 2024). Broad discussions of acute kidney injury further emphasize that ferroptosis is a disease-relevant injury pathway, reinforcing the need for local activation rather than unrestricted systemic induction (Song et al., 2025). The bio-nano interface also complicates translation because protein corona formation, immune clearance and material-tissue interactions can alter the behavior predicted from simplified *in vitro* systems (Björnmalm et al., 2017).

## 3 Nanoplatforms for ferroptosis induction

Ferroptosis-inducing nanoplatforms can be broadly classified into two categories based on their mechanism of action. The first category comprises nanoplatforms that possess intrinsic ferroptosis-inducing activity through their material composition, such as iron-based metal-organic frameworks (MOFs), nanozymes with Fenton-like catalytic activity, and multi-metal nanoparticles that supply catalytic iron or remodel the redox environment. The second category encompasses nanocarrier systems designed to deliver small-molecule ferroptosis inducers, including erastin and its derivatives, RSL3, sorafenib, and other inhibitors targeting GPX4, SLC7A11, or parallel defense pathways. These nanocarriers, typically lipid-based, polymeric, or inorganic systems, improve the solubility, stability, pharmacokinetics, and tumor-targeting efficiency of these otherwise poorly bioavailable small-molecule agents. This section systematically reviews both categories, beginning with intrinsic ferroptosis-inducing nanoplatforms followed by nanocarrier-based delivery systems for small-molecule inducers.

### 3.1 Material-based intrinsic ferroptosis-inducing nanoplatforms

Direct ferroptosis induction by nanomedicine can be organized around two engineering principles. The first increases oxidative pressure through iron supply, Fenton-like catalysis or nanozyme activity. The second weakens antioxidant defenses through GSH depletion, GPX4 or solute carrier family 7 member 11 (SLC7A11) suppression, and interference with parallel defense pathways (Figure 2; Table 1).

**FIGURE 2:**
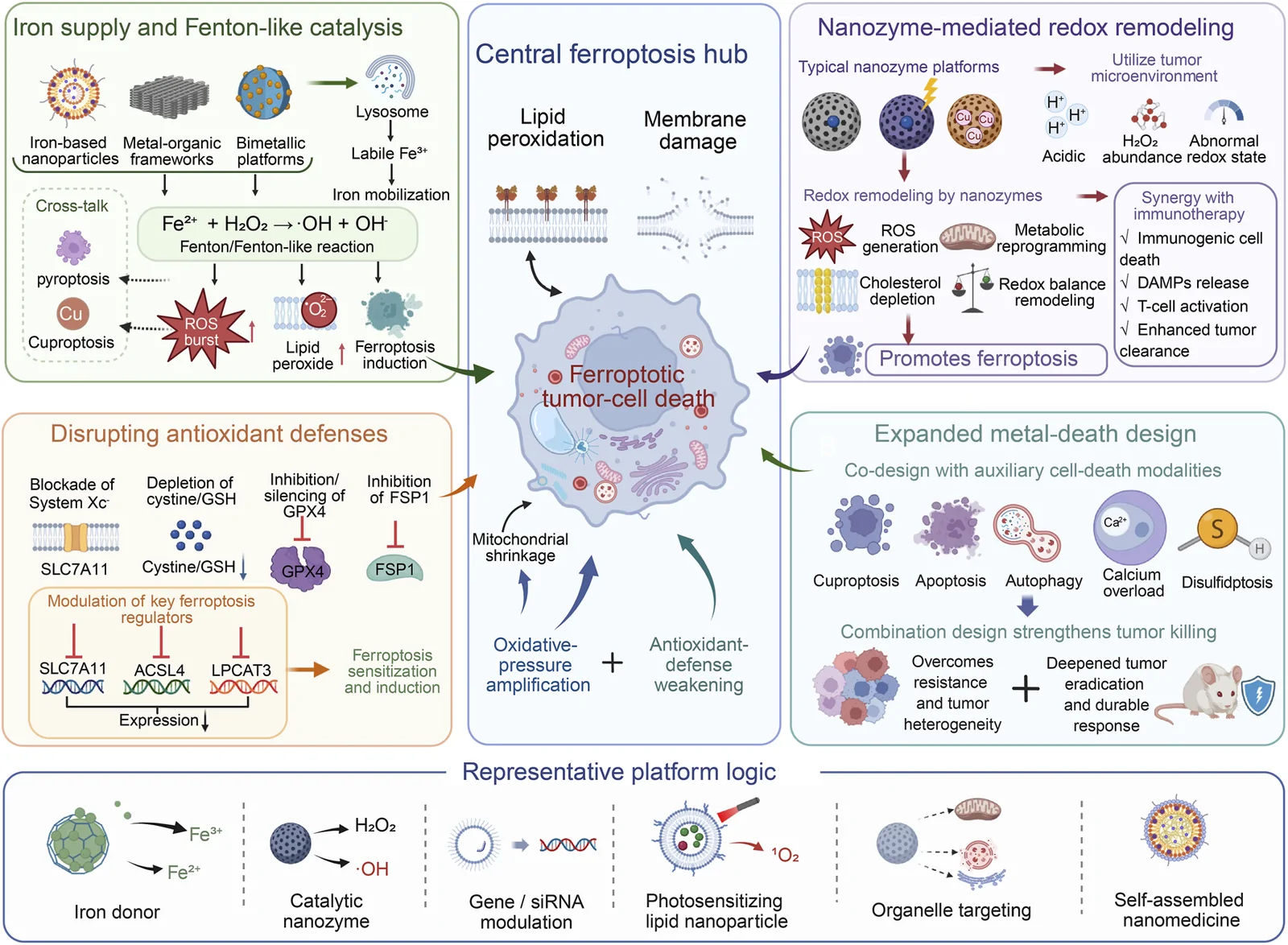
Engineering routes for direct ferroptosis induction by nanoplatforms. The figure shows intrinsic ferroptosis-inducing platforms (iron supply, Fenton-like catalysis, nanozyme redox remodeling, GSH-GPX4-SLC7A11 disruption) and nanocarrier-based delivery of small-molecule inducers (erastin, RSL3, sorafenib), as well as coupling with cuproptosis, pyroptosis, apoptosis or disulfidptosis. The central message is that effective ferroptosis nanomedicine usually combines oxidative-pressure amplification with antioxidant-defense weakening.

**TABLE 1:** Nanoplatform mechanisms for direct ferroptosis induction.

Cancer type	Nanoplatform or intervention	Size	Zeta potential	Drug loading mechanism	Target/mechanism	Core finding	Evidence basis	Ref.
Solid tumors	Iron-mediated lysosomal programming	Not specified	Not specified	Not applicable (intrinsic activity)	Endogenous iron/LMP	Dual ferroptosis/pyroptosis induction	Tumor models	(Zhu et al., 2025a)
Solid tumors	Engineered nanozyme	Not specified	Not specified	Not applicable (intrinsic activity)	Cholesterol depletion	Enhanced ferroptosis and immunotherapy	Preclinical tumor models	(Bai et al., 2024)
Solid tumors	Lactate-regulating nanosystem	Not specified	Not specified	Not applicable (intrinsic activity)	Lactate metabolism; metal death	Synergized cuproptosis and ferroptosis	Preclinical tumor models	(Zhi et al., 2026)
Solid tumors	Mitochondria-targeted nanozyme	∼10 nm	Not specified	Peptide-engineered; mitochondria targeting	Metal homeostasis; mitochondrial stress	Multi-pathway tumor therapy	Preclinical tumor models	(Liu et al., 2025b)
Solid tumors	CRISPR-Cas9 nanotherapy	Not specified	Not specified	GSH/pH-responsive degradation	Gene editing plus metal catalysis	Strengthened cuproptosis/CDT/ferroptosis	Preclinical tumor models	(Wu et al., 2024)
Solid tumors	Ferroptosis assassinates tumor platform	Not specified	Not specified	Not specified	Ferroptosis induction	Established nanotherapy concept	Preclinical tumor models	(Luo et al., 2022)
Solid tumors	Iron-based MOF	∼100–200 nm	Not specified	*In situ* self-assembly	Catalase-like activity; hypoxia relief	Improved PDT/ferroptosis/starvation therapy	Preclinical tumor models	(Chen et al., 2024d)
Bladder cancer	Bimetallic nanozyme	Not specified	Not specified	GOx and PEG integrated into framework	Dual ferroptosis/cuproptosis	Potentiated immunotherapy	Preclinical tumor models	(Chen et al., 2025a)
Lung cancer	Copper-doped polydopamine nanoparticles	Not specified	Not specified	GSH-responsive polydopamine-based hybrid	GSH/GPX4 depletion	Sensitized tumors to checkpoint blockade	Preclinical tumor models	(Jiang et al., 2025)
Oral squamous cell carcinoma	Electro-activated nanozyme	Not specified	Not specified	Not applicable (intrinsic activity)	Multi-enzyme catalysis	Supported *in situ* tumor vaccination	Preclinical tumor models	(Dai et al., 2025)
Glioblastoma	Carrier-free nano-sonosensitizer	Not specified	Not specified	Self-assembly	Sonodynamic metal death	Amplified cuproptosis/ferroptosis	Preclinical tumor models	(Zhu et al., 2024)
Hepatocellular carcinoma	Sorafenib plus FSP1 inhibitor co-delivery	Not specified	Not specified	GSH-responsive polymeric core	Dual ferroptosis defense blockade	Targeted tumor cells and macrophages	Mouse HCC models	(Tang et al., 2025b)
Solid tumors	Single-atom nanozyme	∼50 nm	Not specified	DHA loaded via coordination	Dihydroartemisinin delivery; CDT	Self-enhanced ferroptosis	Preclinical tumor models	(Zhang et al., 2025)
Solid tumors	Photosensitizing lipid nanoparticles	Not specified	Not specified	Not applicable (intrinsic activity)	GPX4 silencing	Enhanced PDT through ferroptosis	Preclinical tumor models	(Bae et al., 2026)
Solid tumors	pH-responsive nanocomposite	Not specified	Not specified	Not specified	SLC7A11/ACSL4/LPCAT3 modulation	Combined sonodynamic immunotherapy	Preclinical tumor models	(Bai et al., 2025c)
Solid tumors	Autophagy-enhancing nanomedicine	Not specified	Not specified	Not applicable (intrinsic activity)	Autophagy-ferroptosis coupling	Enhanced cancer-cell ferroptosis	Preclinical tumor models	(Yang et al., 2021)
Breast cancer	Mitochondria-targeted microneedles	Not specified	Not specified	Spatially targeted delivery	Apoptosis-ferroptosis synergy	Reversed doxorubicin resistance	Drug-resistant tumor models	(Hao et al., 2025)
Solid tumors	Erastin-loaded liposomes	∼100 nm	Positive surface charge	Passive encapsulation; EPR-mediated targeting	Solubility enhancement; GPX4/SLC7A11 inhibition	Enhanced antitumor efficacy with reduced systemic toxicity	Preclinical tumor models	(Xu Y. et al., 2026)
Hepatocellular carcinoma	Sorafenib-loaded PLGA nanoparticles	175–225 nm	−10 to −27 mV	Encapsulation during nanoprecipitation	Drug delivery; ferroptosis induction	Enhanced ferroptosis and antitumor activity	HCC models	(Tang B. et al., 2025, Xuan et al., 2025)
Glioblastoma	Erastin-loaded mesoporous silica nanoparticles	∼200 nm	−17.8 mV	Pore loading	Targeted delivery; blood-brain barrier penetration	Improved tumor retention and ferroptosis induction	GBM models	(Yang C. et al., 2024, Yang F. et al., 2024)

#### 3.1.1 Iron supply and fenton-like catalysis

Iron-centered nanoplatforms use the chemical foundation of ferroptosis as a therapeutic advantage. Programmed enhancement of endogenous iron-mediated lysosomal membrane permeabilization induced dual ferroptosis and pyroptosis in tumors, illustrating how iron mobilization can be coupled to multiple death programs (Zhu L. et al., 2025). Engineered nanozymes that deplete cholesterol can enhance ferroptosis and immunotherapy, linking membrane composition, oxidative injury and immune response in one design (Bai et al., 2024). A lactate metabolism-regulating nanosystem combined cuproptosis and ferroptosis to enhance cancer immunotherapy, showing that metabolic background can be built into metal-dependent death strategies (Zhi et al., 2026).

Beyond direct iron delivery, intracellular iron mobilization via autophagy—specifically ferritinophagy, the selective autophagic degradation of ferritin—has emerged as an alternative strategy to amplify the labile iron pool and potentiate Fenton-driven lipid peroxidation. Nanomedicine-enabled ferritinophagy has been shown to enhance cancer-cell ferroptosis, supporting the concept that intracellular degradation pathways can be harnessed to increase ferroptotic pressure through iron redistribution.

Metal-organic frameworks and multi-metal platforms provide structural routes for controlling catalytic activity and cargo release. A mitochondria-targeted nanozyme platform regulated ferroptosis and cuproptosis for multipathway tumor therapy, positioning organelle targeting as a way to focus metal-induced stress (Liu C. et al., 2025). A CRISPR-Cas9 gene-editing strategy strengthened cuproptosis, chemodynamic therapy and ferroptosis, showing that nanomaterial catalysis can be combined with genetic intervention to intensify metal-dependent death (Wu et al., 2024). An iron-based metal-organic framework with catalase-like activity improved photodynamic, ferroptotic and starvation therapy by reversing tumor hypoxia, highlighting the importance of oxygen handling in ROS-dependent ferroptosis therapy (Chen Y. et al., 2024). A bimetallic nanozyme induced dual ferroptosis and cuproptosis and potentiated immunotherapy in bladder cancer, reinforcing the value of metal combinations for amplifying oxidative and immune effects (Liu C. et al., 2025).

#### 3.1.2 Nanozyme-mediated redox remodeling

Nanozymes are particularly relevant to ferroptosis because they can turn endogenous TME chemistry into therapeutic oxidative stress. A single-atom nanozyme delivered dihydroartemisinin for self-enhanced chemodynamic therapy and ferroptosis, showing how catalytic activity and drug action can reinforce each other (Zhang et al., 2025). An electro-activated nanozyme promoted *in situ* tumor vaccination through multi-enzyme catalysis and electrodynamic therapy, linking redox catalysis to immune stimulation (Dai et al., 2025). Copper-doped polydopamine nanoparticles depleted GSH and GPX4 while sensitizing lung tumors to checkpoint blockade, indicating that redox remodeling can support both ferroptosis and immunotherapy (Jiang et al., 2025). Beyond conventional nanozyme designs, metal–phenolic networks-based nanozymes have recently emerged as a versatile platform that simultaneously reprograms tumor metabolism and immunosuppressive microenvironments to amplify ferroptosis and potentiate antitumor immunity, further expanding the catalytic and immunomodulatory capabilities of nanozyme-mediated ferroptosis therapy (Ma et al., 2026).

The translational appeal of nanozymes lies in their ability to operate in abnormal tumor chemistry. Tumors often contain elevated hydrogen peroxide, altered pH and high GSH, and catalytic nanomaterials can convert these features into selective redox injury. Yet the same catalytic reactivity creates a safety burden because off-target ROS generation may injure normal tissues if biodistribution and degradation are not controlled.

#### 3.1.3 Disrupting antioxidant defenses

Defense-disruption strategies address a central limitation of iron-supply approaches: tumor cells can buffer lipid peroxidation. Co-delivery of sorafenib and an FSP1 inhibitor triggered dual ferroptosis in tumor cells and immunosuppressive macrophages in hepatocellular carcinoma models, suggesting that simultaneous targeting of tumor and immune compartments can strengthen ferroptosis-based immunotherapy (Tang C. et al., 2025). Photosensitizing lipid nanoparticles enhanced photodynamic cancer therapy through GPX4 silencing, showing that genetic suppression of a ferroptosis checkpoint can amplify ROS-based treatment (Bae et al., 2026). A pH-responsive nanocomposite combined sonodynamic immunotherapy with ferroptosis and calcium overload through SLC7A11, acyl-CoA synthetase long-chain family member 4 (ACSL4) and lysophosphatidylcholine acyltransferase 3 (LPCAT3) pathway modulation, illustrating how ferroptosis can be integrated with ion stress and immune activation (Bai X. et al., 2025).

Collectively, these studies support defense disruption as a necessary complement to catalytic iron strategies, especially in tumors with high antioxidant buffering.

Additional metal- and redox-active platforms broaden this mechanism class across disease. Antibody-functionalized iron nanoplatforms, senescence-primed metal-organic frameworks, SLC7A11/GPX4-targeted nano-MOFs and albumin nanoparticles all reinforce the idea that catalytic or carrier structure can be adapted to tumor-specific ferroptosis vulnerability (Bai X. et al., 2025; Gao et al., 2025; Huang et al., 2025; Xu S. et al., 2025). GSH-consuming polymeric nanoparticles, hybrid nanoenzymes, labile-iron chemodynamic inducers and multifunctional metal-coordinated platforms extend this principle by combining antioxidant depletion with iron or peroxide supply (Chen X. et al., 2026; Pei et al., 2025; Wei P. et al., 2025; Zhou et al., 2025). Newer bimetallic, CuFeTe_2_, metal-phenolic and glutamine-metabolism-intervening platforms further show that ferroptosis can be co-designed with cuproptosis, apoptosis, lipophagy or immune activation rather than treated as an isolated death mode (Ding J. et al., 2026; Liu M. et al., 2026; Liu T. et al., 2026; Wang Y. et al., 2025).

### 3.2 Nanocarriers for small-molecule ferroptosis inducers

Although intrinsic ferroptosis-inducing nanoplatforms offer a “carrier-free” approach, many classical ferroptosis-inducing small molecules—including erastin, imidazole ketone erastin (IKE), RSL3, ML162, and sorafenib—suffer from poor aqueous solubility, rapid clearance, off-target toxicity, and limited tumor accumulation (Liang et al., 2019). Nanocarrier-based delivery systems have been extensively explored to overcome these pharmacokinetic barriers while enabling tumor-selective ferroptosis induction.

Lipid-based nanocarriers represent a promising platform for the delivery of ferroptosis inducers. For example, folic acid-modified fluoro-liposomes loaded with erastin improved the oral bioavailability and tumor-targeting capacity of erastin and effectively suppressed tumor growth *in vivo* (Hong et al., 2025). Similarly, RSL3-encapsulated lipid nanoparticles achieved sustained GPX4 inhibition and potent ferroptosis induction in drug-resistant cancer cells (Habib et al., 2025). Hybrid liposomal systems co-delivering ferroptosis inducers with chemotherapeutics or photosensitizers have further demonstrated synergistic antitumor effects through concurrent blockade of multiple survival pathways (Li J. et al., 2025).

Polymeric nanoparticles offer complementary advantages, including high drug-loading capacity, tunable release kinetics, and facile surface functionalization for active targeting. Sorafenib-loaded PEGylated poly(lactic-co-glycolic acid) (PLGA) nanoparticles have been shown to enhance ferroptosis induction in hepatocellular carcinoma through combined drug release and iron-dependent oxidative stress (Liu et al., 2021). GSH-responsive polymeric nanocarriers have been engineered to release GPX4 inhibitors specifically in the reducing tumor microenvironment, thereby improving therapeutic index (Xie et al., 2026).

Inorganic nanocarriers, particularly mesoporous silica nanoparticles (MSNs) and gold nanoparticles, have also been explored for ferroptosis inducer delivery. MSNs functionalized with tumor-homing ligands have demonstrated efficient delivery of erastin and RSL3 to glioblastoma and ovarian cancer models, with enhanced blood-brain barrier penetration and prolonged tumor retention (Cao et al., 2023; Chen S. et al., 2024). Iron-oxide nanoparticles have been repurposed as dual-function platforms combining magnetic hyperthermia with sorafenib delivery, enabling ferroptosis amplification through both chemical and physical stimuli (Tang et al., 2024).

A growing trend in this domain is the design of nanocarriers that co-deliver small-molecule ferroptosis inducers with additional therapeutic agents, such as checkpoint inhibitors, photosensitizers, or chemotherapeutics, to achieve multimodal ferroptosis-based therapy. For example, co-delivery of sorafenib and an FSP1 inhibitor in polymeric nanoparticles triggered dual ferroptosis in both tumor cells and immunosuppressive macrophages, substantially enhancing immunotherapy efficacy in hepatocellular carcinoma (Tang C. et al., 2025). Despite these advances, the clinical translation of nanocarrier-based ferroptosis inducer delivery faces challenges related to batch-to-batch reproducibility, carrier-induced immunogenicity, and the need for robust pharmacodynamic biomarkers to confirm target engagement in patients.

## 4 Precision delivery and synergistic therapy

The second major task for ferroptosis nanomedicine is to deliver and activate ferroptosis pressure in the right place. Precision delivery, TME-responsive activation and combination therapy are not separate design philosophies. They are interdependent solutions to the same problem: ferroptosis must be strong enough inside tumors while remaining controlled in normal tissues (Figure 3; Table 2).

**FIGURE 3:**
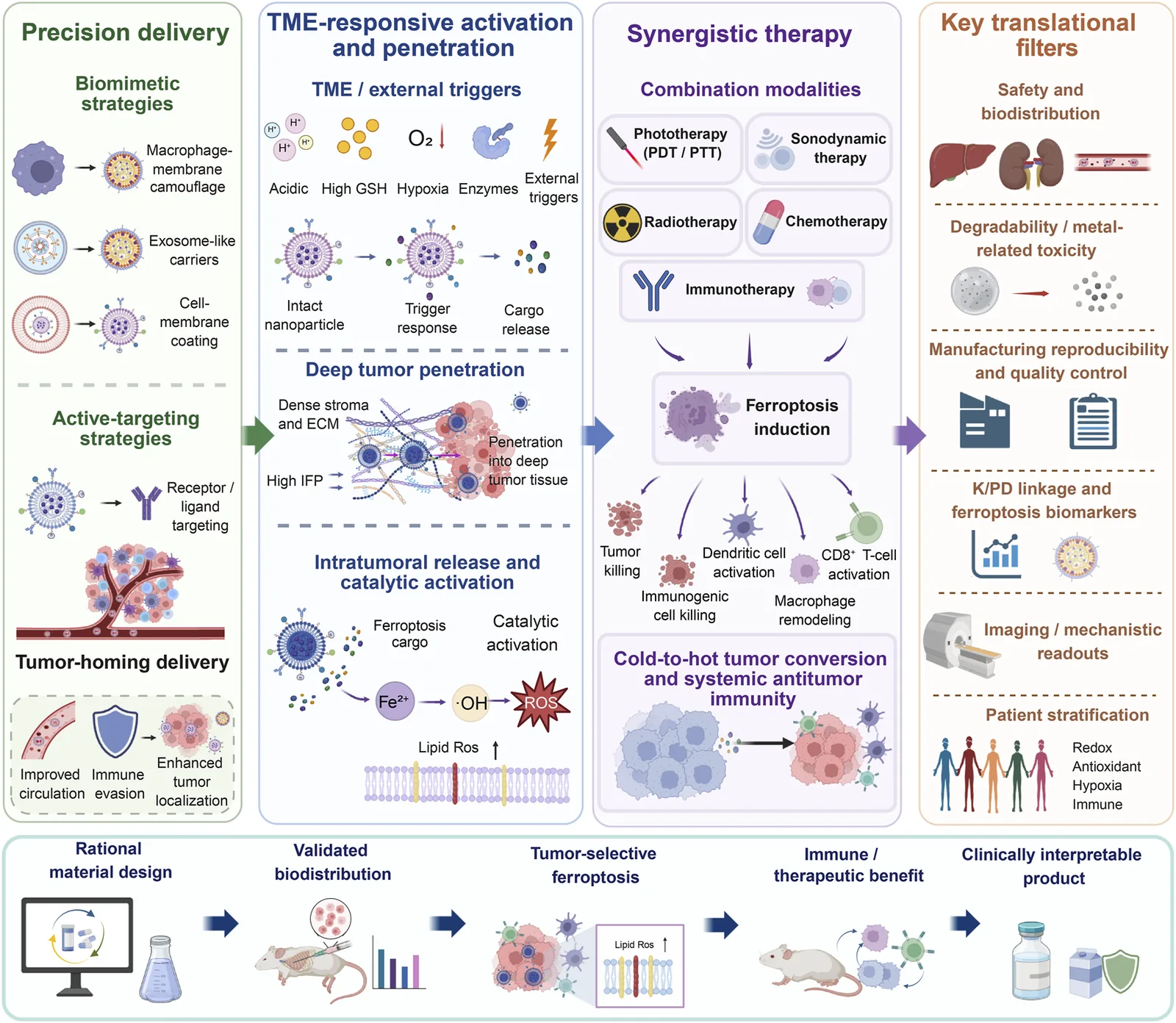
Precision delivery and synergistic therapy. The figure integrates biomimetic delivery, active targeting, TME-responsive release, phototherapy, sonodynamic therapy, radiotherapy, chemotherapy and immunotherapy. These can improve tumor-localized ferroptosis while reducing the dependence on passive accumulation alone.

**TABLE 2:** Synergistic ferroptosis nanomedicine strategies in cancer.

Cancer type	Nanoplatform type	Combination strategy	Mechanistic rationale	Therapeutic effect	Evidence basis	Ref.
Glioblastoma	Macrophage membrane-camouflaged nanoparticle	Macrophage membrane camouflage + ferroptosis induction	Mitochondrial damage via iron overload and ROS; biomimetic membrane enhances BBB penetration	Induced tumor ferroptosis; prolonged survival in orthotopic GBM models	Preclinical GBM models	(Cao et al., 2023)
Solid tumors	Biomimetic nanoplatform	Sonodynamic therapy + ferroptosis + CD47 checkpoint blockade	SDT-generated ROS induces ferroptotic lipid peroxidation; CD47 blockade enhances phagocytic clearance of tumor cells by macrophages	Enhanced antitumor immunity and prolonged survival	Preclinical tumor models	(Liao et al., 2025)
Glioblastoma	Tumor exosome-based nanocarrier	Ferroptosis + apoptosis induction via drug-loaded exosomes	Endogenous exosomes cross BBB; dual death pathway activation overcomes resistance	Improved tumor-directed delivery and antitumor efficacy	Preclinical GBM models	(Xu et al., 2026b)
Prostate cancer	Genetically engineered membrane-coated nanoparticle	PSMA receptor targeting + ferroptosis induction	PSMA-mediated active targeting increases tumoral accumulation; GPX4 downregulation enhances ferroptosis	Suppressed CRPC tumor growth in mouse models	CRPC models	(Li et al., 2024c)
Hepatocellular carcinoma	Biomimetic nanoparticle (lenvatinib + FePt)	Lenvatinib + FePt co-delivery + ferroptosis/apoptosis	Lenvatinib inhibits VEGF receptors; FePt amplifies ferroptosis via Fenton reaction; synergistic apoptosis	Enhanced antitumor effect in HCC xenografts	HCC models	(Xuan et al., 2025)
Hepatocellular carcinoma	RRM2-targeted nanocarrier	RRM2 silencing + RFA + ferroptosis induction	RRM2 inhibition increases lipid peroxidation and sensitizes tumor cells to RFA-induced ferroptosis; immune remodeling	Enhanced RFA efficacy and local tumor control	HCC models	(Hou et al., 2025)
Colorectal cancer	Nano-herb delivery system	pH/GSH-responsive CO-driven delivery + ferroptosis + immunotherapy	CO gas therapy promotes mitochondrial dysfunction; pH/GSH responsiveness enables tumor-specific release	Enhanced immunotherapy in CRC models	CRC models	(Yang et al., 2024a)
Solid tumors	Self-enriching nanozyme	Photothermal cascade amplification + ferroptosis + photoacoustic imaging	Near-infrared irradiation induces photothermal effect; nanozyme amplifies ROS generation and ferroptosis; self-enriching improves tumor accumulation	Synergistic therapy and real-time imaging	Preclinical tumor models	(Zhu et al., 2025b)
Solid tumors	NIR-II conjugated polymer phototheranostic	NIR-II phototheranostics + hyperthermia + ferroptosis + immunotherapy	NIR-II hyperthermia increases tumor blood flow and drug penetration; promotes GPX4 inactivation	Improved immunotherapy and tumor regression	Preclinical tumor models	(Chen et al., 2025b)
Colorectal cancer	Fe3O4 photothermal nanoparticle	Photothermal therapy + immunogenic ferroptosis	Fe3O4-mediated PTT induces heat stress; iron release triggers ferroptosis; DAMPs activate antitumor immunity	Synergistic tumor ablation and immune activation	CRC models	(Bae et al., 2026)
Solid tumors	Hybrid liposome	PDT + chemotherapy + ferroptosis	Photosensitizer-generated ROS and chemotherapeutics synergistically disrupt redox homeostasis; GSH depletion enhances ferroptosis	Multimodal tumor killing and reduced systemic toxicity	Preclinical tumor models	(Huang et al., 2024)
Solid tumors	Osmium-ferritin nanoplatform	PDT/PTT switching + ferroptosis + immunotherapy	Osmium complex enables photodynamic-photothermal switching; ferritin provides iron for ferroptosis; ICD induces antitumor immunity	Reprogrammed tumor immunity and abscopal effect	Preclinical tumor models	(Liao et al., 2025)
Solid tumors	Peptide nanoassembly	Peptide nanoassembly + paclitaxel + ferroptosis	Self-assembling peptides sensitize drug-resistant cells; paclitaxel stress converted into ferroptotic vulnerability via lipid peroxidation	Overcame paclitaxel resistance; tumor regression	Paclitaxel-resistant models	(Lin et al., 2025)
Solid tumors	Chemo-photothermal nanoplatform	Chemotherapy + photothermal therapy + ferroptosis + immunotherapy	Chemo-drug and PTT-induced heat stress synergistically increase ROS; ferroptotic cell death releases DAMPs	Ignited antitumor immunity; eliminated primary and distant tumors	Preclinical tumor models	(Lin et al., 2026)
Hepatocellular carcinoma	Fe(III)-COF-sorafenib nanoplatform	Sorafenib delivery + chemo-ferroptosis + immunotherapy	Sorafenib inhibits system Xc−; Fe(III)-COF provides Fenton-reactive iron; ICD promotes immune cell infiltration	Enhanced HCC immunotherapy; tumor microenvironment remodeling	HCC models	(Bai et al., 2025a)
Solid tumors	Dual-regulated death nanoparticles	Ferroptosis + cuproptosis co-induction	Cu-based nanoparticles induce cuproptosis via mitochondrial Fe-S protein disruption; ferroptosis is amplified by GSH depletion and GPX4 inactivation	Potentiated cancer immunotherapy and tumor suppression	Preclinical tumor models	(Li Y. et al., 2024)
Solid tumors	Bimetallic peroxide nanoparticles	Ferroptosis + cuproptosis + cGAS-STING activation	Bimetallic peroxide releases Fe and Cu ions; Fe-catalyzed Fenton reaction and Cu-induced cuproptosis; cGAS-STING activates innate immunity	Enhanced tumor immunotherapy and durable antitumor responses	Preclinical tumor models	(Liu et al., 2025a)
Solid tumors	Glycyrrhizic acid nanoplatform	Ferroptosis induction + HMGB1 blockade	Ferroptotic cell death releases HMGB1; glycyrrhizic acid binds HMGB1 to modulate inflammatory response and prevent excessive immune activation	Balanced immunotherapy efficacy and reduced toxicity	Preclinical tumor models	(Sha et al., 2025)
Prostate cancer	Transformable supramolecular peptide assemblies	Self-enhanced ferroptosis + cancer immunotherapy	Peptide assemblies undergo TME-triggered morphological transformation; enhanced ROS generation and ferroptosis; promotes dendritic cell maturation	Primed cancer immunotherapy in cold tumors; improved T-cell infiltration	Cold tumor models	(Wang et al., 2024)

### 4.1 Biomimetic and active targeting

Biomimetic delivery attempts to make nanosystems behave more like biological entities. Macrophage membrane-camouflaged nanoparticles induced ferroptosis by promoting mitochondrial damage in glioblastoma, demonstrating how membrane camouflage can improve delivery in a tumor type with formidable anatomical barriers (Cao et al., 2023). A sonodynamic therapy-boosted biomimetic nanoplatform targeted ferroptosis and cluster of differentiation 47 (CD47) vulnerabilities for cancer immunotherapy, linking membrane-based targeting with immune checkpoint-related remodeling (Liao et al., 2025). Tumor exosome-based drug delivery targeted ferroptosis and apoptosis for glioblastoma therapy, showing that endogenous vesicle-like carriers can be used to cross delivery barriers and combine death programs (Xu Y. et al., 2026).

Active targeting can further refine the site of ferroptosis induction. Genetically engineered membrane-coated nanoparticles enhanced prostate-specific membrane antigen (PSMA) membrane antigen targeting and ferroptosis treatment of castration-resistant prostate cancer, supporting receptor-guided ferroptosis delivery in a defined tumor microenvironment t (Li et al., 2024c). Biomimetic co-delivery of lenvatinib and FePt nanoparticles enhanced ferroptosis and apoptosis in hepatocellular carcinoma, illustrating how targeted co-delivery can address both kinase-driven survival and redox vulnerability (Xuan et al., 2025). An RRM2-targeted nanocarrier enhanced radiofrequency ablation efficacy in hepatocellular carcinoma through ferroptosis amplification and immune remodeling, linking active targeting with local therapy sensitization (Hou et al., 2025).

### 4.2 TME-responsive release and penetration

TME-responsive systems use tumor chemistry as an activation signal. A pH- and GSH-responsive carbon monoxide-driven nano-herb delivery system enhanced immunotherapy in colorectal cancer, indicating that pH and redox triggers can coordinate release with immune effects (Yang C. et al., 2024). A self-enriching nanozyme with photothermal-cascade amplification provided TME-responsive synergistic therapy and photoacoustic imaging, showing how accumulation, activation and imaging can be integrated (Zhu X. et al., 2025). Near-infrared II (NIR-II) conjugated polymer phototheranostics enhanced ferroptosis and immunotherapy through hyperthermia, connecting optical activation with redox and immune modulation (Chen et al., 2025b).

Penetration remains a distinct obstacle because tumor accumulation does not guarantee access to deep tumor regions. Manganese-induced photothermal-ferroptosis therapy addressed physical barriers such as fibrotic matrix and elevated interstitial pressure, showing that stromal environment can limit ferroptosis nanomedicine performance (Chen G. et al., 2024). A nanoplatform that reversed tumor hypoxia improved photodynamic, ferroptotic and starvation therapy, emphasizing that oxygenation and penetration constraints shape ROS-dependent outcomes. A topology-regulated nanocatalyst enabled ferroptosis-mediated cancer phototherapy, suggesting that material structure can tune activatable catalytic function (Chen K. et al., 2024). Beyond conventional hypoxia relief strategies, alloy nanosheets with multi-enzyme-mimicking activities—incorporating both Pd and Fe—have been shown to alleviate hypoxia-induced ferroptosis resistance and immunosuppression, thereby priming the cancer-immunity cycle and achieving enhanced NIR-II photoimmunotherapy, illustrating how addressing hypoxia can simultaneously overcome ferroptosis resistance and immune suppression (Chen et al., 2025c).

### 4.3 Energy-assisted ferroptosis

Photodynamic therapy (PDT), photothermal therapy (PTT), sonodynamic therapy and radiation can intensify ferroptosis by increasing ROS, heat stress or membrane injury. Photodynamic-photothermal switching in an osmium-ferritin nanoplatform activated near-infrared-driven tumor immunity reprogramming, showing that optical control can combine local damage with immune modulation (Liao et al., 2025). Photothermal Fe_3_O_4_ nanoparticles induced immunogenic ferroptosis for synergistic colorectal cancer therapy, linking PTT, iron-based ferroptosis and immune activation (Li et al., 2024b). The rational design of photothermal nanoplatforms for immunotherapy has been systematically reviewed from a multidimensional perspective, providing valuable guidance for integrating photothermal effects with ferroptosis induction (Wei et al., 2024). Hybrid liposomes designed for PDT, chemotherapy and ferroptosis disrupted redox homeostasis, supporting multimodal ROS amplification (Huang et al., 2024).

Sonodynamic and local energy approaches extend this logic beyond light. Carrier-free nano-sonosensitizers amplified cuproptosis and ferroptosis in glioblastoma, indicating that ultrasound-responsive platforms can intensify metal-dependent death in difficult tumor settings (Zhu et al., 2024). A biomimetic nanosystem enhanced prostate cancer radiotherapy by inducing ferroptosis, demonstrating that ferroptosis can act as a radiosensitizing mechanism (Hou et al., 2025). These energy-assisted approaches are attractive because they can add spatial control, but they also require careful matching between tumor location, penetration depth and external activation.

The delivery study also shows rapid diversification beyond simple encapsulation. Biomimetic iridium nanozymes, biomimetic photodynamic nanoparticles, platelet membrane-engineered PDT platforms and cell membrane-engineered FePDA nanoparticles combine membrane-like targeting with ferroptosis or pyroptosis-associated immune activation (Chen C. et al., 2026; Ding L. et al., 2026; Hua et al., 2026; Lu et al., 2026). Cancer cell membrane/iRGD liposomes, CXCR4-engineered fibroblast membrane nanovesicles, dual-targeted biomimetic nanoparticles and outer membrane vesicle-coated ferrocene nanoparticles illustrate how homing ligands, engineered membranes and microbial vesicle concepts can be used to improve tumor-localized ferroptosis therapy (Bai L. et al., 2025; Guo et al., 2026; Shen et al., 2025; Zu et al., 2025).

Emerging responsive platforms add external control and microenvironment selectivity. Dual-targeting bimetallic nanozymes, mitochondrion-targeted NIR-II modulators, photothermal-responsive multi-enzyme nanoprobes and targeted GPX4-degradation nano-PDTACs connect imaging, light activation, antioxidant depletion and ferroptosis-immunotherapy into integrated therapeutic systems (He et al., 2025; Ji et al., 2026; Li C. et al., 2025; Wang M. et al., 2025). Ultrasound-responsive biomimetic nanocarriers, low-dose X-ray-activated radiodynamic platforms, piezocatalytic magnesium-releasing systems and pH-responsive antibacterial Fe^3+^ nanoparticles show that ultrasound, X-rays, mechanical catalysis and intratumoral bacteria can also be used as ferroptosis-amplifying environment (Liao et al., 2026; Song et al., 2026; Sun et al., 2026; Yu et al., 2026).

### 4.4 Chemo-ferroptosis and immune synergy

Chemo-ferroptosis strategies exploit the fact that chemotherapy often induces stress states that can be converted into ferroptotic vulnerability. A peptide-based nanoassembly enhanced ferroptosis to overcome paclitaxel resistance, showing how nanomedicine can convert resistance-associated stress into therapeutic susceptibility (Lin et al., 2025). Chemo-photothermal synergy ignited antitumor immunity through ferroptosis, illustrating how conventional chemotherapy can be integrated with heat and redox injury (Lin et al., 2026). An Fe(III)-covalent organic framework-sorafenib nanoplatform induced chemo-ferroptosis and enhanced hepatocellular carcinoma immunotherapy, supporting a direct connection between kinase inhibition, ferroptosis and immune response (Bai B. et al., 2025).

Spatially targeted delivery strategies have further expanded the scope of chemo-ferroptosis combination. Mitochondria-targeted microneedles reversed doxorubicin resistance by combining apoptosis and ferroptosis, demonstrating that subcellular organelle targeting can potentiate chemotherapy efficacy through concurrent activation of complementary death programs (Hao et al., 2025).

The immunological consequences of ferroptosis have become a major reason for using nanomedicine platforms. The synergistic potential of nanoparticle-enabled ferroptosis-immunotherapy has been comprehensively outlined, advancing multidimensional nanoplatform design principles that integrate ferroptosis-inducing agents with immunomodulatory therapeutics (Wei X. et al., 2025). Nanoparticles that synergized ferroptosis and cuproptosis potentiated cancer immunotherapy, indicating that regulated death combinations can enhance immune responses (Li Y. et al., 2024). Bimetallic peroxide nanoparticles induced ferroptosis and cuproptosis and activated the cyclic GMP-AMP synthase-stimulator of interferon genes (cGAS-STING) pathway for enhanced tumor immunotherapy, linking metal peroxide chemistry with innate immune signaling (Liu B. et al., 2025). A glycyrrhizic acid-loaded nanoplatform combined ferroptosis induction with high-mobility group box 1 (HMGB1) blockade, showing that the immunological by-products of ferroptosis may need to be tuned rather than simply amplified (Sha et al., 2025). Transformable supramolecular self-assembled peptides primed ferroptosis for cancer immunotherapy, supporting the broader idea that material transformation can coordinate cell death and immune activation (Wang et al., 2024). These studies suggest that ferroptosis nanomedicine should be evaluated not only by tumor-cell killing but also by its effects on antigen presentation, macrophage polarization, T-cell infiltration and checkpoint responsiveness.

The immunotherapy-oriented study is also expanding toward metabolic, antigenic and stromal rewiring. Biomimetic platforms that activate cGAS-STING, major histocompatibility complex class I (MHC-I) membrane-encapsulated copper/ferrihydrite systems, melanoma lipid-metabolism disruption platforms and FABP5/STING-targeted ablation sensitizers all point to ferroptosis as a bridge between oxidative death and immune recognition (Peng H. et al., 2025; Tang B. et al., 2025; Yang M. D. et al., 2025; Yang R. et al., 2025). Photothermal-chemotherapy/ferroptosis platforms, fenbendazole plus photothermal therapy, GSH-responsive photosensitizer systems and tumor-targeted tRF therapeutics further suggest that ferroptosis nanomedicine can be tuned through drug repurposing, lipid metabolism, system Xc-inhibition and photosensitizer chemistry (Lin et al., 2026; Ouyang et al., 2026; Peng B. et al., 2025; Xu X. et al., 2026).

## 5 Clinical translation and future design

Ferroptosis nanomedicine has produced a large and creative preclinical study, but clinical progress requires a more restrictive translational filter. A platform must be effective, interpretable, manufacturable, degradable and monitorable. These requirements are especially important for metal-containing and catalytic materials, where the therapeutic mechanism and potential toxicity can arise from the same chemical property.

### 5.1 Safety and biodistribution

Long-term biodistribution and degradation are central issues for ferroptosis nanomedicine. Metal-based nanoparticles can raise cardiovascular toxicity concerns, which is relevant because many ferroptosis platforms use iron, copper, manganese, nickel or composite metal systems (Kim et al., 2025). Visualization of ferroptosis in brain diseases and glioma-directed nanomedicine emphasizes that monitoring ferroptosis *in vivo* remains technically difficult, particularly when the target tissue is protected by specialized barriers (Wang et al., 2023). Ferroptosis nanomedicine reviews have therefore called for stronger safety assessment, biodistribution analysis and standardized evaluation of metabolic and immunological consequences.

The same ability to generate ROS, release metal ions or deplete GSH may damage normal organs if tumor selectivity is insufficient. Studies of ferroptosis-associated acute kidney injury and chemotherapy toxicity support the need to distinguish antitumor ferroptosis from unwanted ferroptosis in healthy tissues. Encouragingly, early-phase clinical data from CNSI-Fe(II) intratumoral injection have demonstrated an acceptable safety profile with no obvious liver, kidney, or hematologic toxicity in 19 treated patients, although dose-limiting toxicity was observed in one subject. Preclinical studies should therefore report not only tumor inhibition but also organ accumulation, clearance, histological injury, inflammatory markers and recovery after treatment.

### 5.2 Manufacturing and pharmacokinetic standards

Cancer nanomedicine translation depends on reproducible particle size, surface chemistry, drug loading, release kinetics, sterility, stability and batch consistency. Bio-nano interface studies show that biological identity can diverge from synthetic identity after exposure to proteins, cells and immune components, which complicates prediction from material characterization alone. EPR variability further means that pharmacokinetic success cannot be inferred from nanoscale formulation alone, because tumor vascular state and tissue pressure strongly influence delivery.

For ferroptosis therapies, pharmacokinetic and pharmacodynamic standards must be linked. Reporting particle dose without iron release, catalytic activity, lipid-peroxidation readouts and tissue exposure is insufficient for comparing platforms. A useful translational package should relate material properties to tumor accumulation, ferroptosis biomarkers, antitumor efficacy and normal-organ safety. Without this linkage, increasingly complex all-in-one platforms may remain difficult to rank, reproduce or regulate.

### 5.3 Biomarkers and patient selection

The field lacks clinically validated biomarkers that can confirm ferroptosis induction in living patients. Mechanistic reviews have proposed that system Xc-, GPX4, lipid metabolism, iron handling and mitochondrial defense pathways may guide nanomedicine design, but these markers are not yet sufficient as standalone clinical selection tools (Zhang et al., 2024). Emerging clinical studies have begun to incorporate pharmacodynamic endpoints, such as tumor SLC7A11 and GPX4 expression, as well as lipid peroxidation markers including 4-hydroxynonenal (4-HNE), to monitor ferroptosis pathway engagement in patients. Ferroptosis imaging and visualization approaches are promising but remain distant from routine patient stratification. Translational reviews also highlight the need to connect ferroptosis biomarkers with immune environment, metabolic state and treatment response rather than measuring isolated pathway components—a principle increasingly supported by early clinical correlative data.

Future ferroptosis nanomedicine should move from universal platform claims toward disease-matched design. Tumors with high antioxidant buffering may require GPX4, GSH or FSP1 suppression. Hypoxic tumors may require oxygen-generating or hypoxia-adaptive platforms. Immunologically cold tumors may benefit from ferroptosis designs that promote antigen release, dendritic-cell activation and checkpoint responsiveness. This patient- and context-aware strategy would make ferroptosis nanomedicine more compatible with precision oncology.

### 5.4 Clinical translation landscape of ferroptosis-based therapies

Despite the rapid expansion of preclinical ferroptosis research, clinical translation remains in its early stages. Nevertheless, several ferroptosis-inducing agents have entered clinical trials, and the first nanomedicine-based ferroptosis inducer has generated preliminary human data.

#### 5.4.1 Ferroptosis-inducing agents in clinical trials

The most clinically advanced ferroptosis-related agent is CNSI-Fe(II) (carbon nanoparticle-loaded iron), an intratumorally injected nanopharmaceutical that delivers Fe^2+^ ions directly into tumor cells to induce ferroptosis (Xie et al., 2026). A first-in-human Phase I dose-escalation trial (NCT06048367) in patients with advanced solid tumors enrolled 19 heavily pretreated patients. The results demonstrated an acceptable safety profile with no obvious liver, kidney, or hematologic toxicity; dose-limiting toxicity was observed in only one subject (90 mg group). Notably, one patient achieved complete tumor response (60 mg group), one achieved partial response (30 mg group), and 11 achieved stable disease, yielding a disease control rate of 87%. Four patients (21.1%) with progressive, treatment-refractory tumors (ovarian serous carcinoma, pancreatic adenocarcinoma, myoepithelioma-like tumor, and thyroid carcinoma) achieved long-term survival of 28.9–36.2 months post-injection, accompanied by restored sensitivity to chemotherapies or targeted agents to which they were previously resistant. A Phase Ib/IIa study is currently planned to further evaluate safety, pharmacokinetics, and efficacy.

Sulfasalazine (SSZ), an FDA-approved anti-inflammatory drug, inhibits SLC7A11 and has been repurposed as a ferroptosis inducer. SSZ is currently being evaluated in a Phase I trial (NCT04205357) as a radiosensitizer combined with stereotactic radiosurgery for recurrent glioblastoma, and a Phase I/II trial for ferroptosis-dependent induction therapy in acute myeloid leukemia is recruiting subjects (NCT05580861) (Skeie et al., 2026). However, a previous Phase I/II trial of SSZ for progressive malignant glioma was terminated prematurely due to lack of efficacy and serious neurological adverse events, underscoring the importance of patient selection and biomarker guidance.

Sorafenib, a multi-kinase inhibitor approved for hepatocellular carcinoma, indirectly promotes ferroptosis by inhibiting SLC7A11-mediated cystine uptake, thereby limiting glutathione synthesis. A Phase II clinical trial confirmed that sorafenib combined with stereotactic body radiotherapy overcame resistance in colorectal cancer liver metastases with high SLC7A11 expression by inducing ferroptosis. In glioblastoma, sorafenib trials have reported median progression-free survival ranging from 1.8 to 7.9 months and overall survival from 4.4 to 18.0 months.

Artesunate, a semisynthetic derivative of the antimalarial drug artemisinin, increases intracellular iron levels and reactive oxygen species, contributing to ferroptosis induction. It has entered early-phase clinical trials in breast cancer and other solid tumors (e.g., NCT00764036, NCT03792516) (Ooko et al., 2015; von Hagens et al., 2017). A Phase II trial evaluating neoadjuvant artesunate in stage II/III colorectal cancer is ongoing (NCT02633098) (Krishna et al., 2015).

APR-246 (eprenetapopt), a first-in-class small molecule that reactivates mutant p53 and targets cellular redox balance, induces both apoptosis and ferroptosis in TP53-mutant cancer cells. Two Phase II trials (NCT03072043, NCT03588078) evaluating APR-246 in combination with azacitidine in TP53-mutant myelodysplastic syndromes and acute myeloid leukemia have reported an overall response rate of 71%, with preclinical models confirming that APR-246 induces ferroptosis and enhances chemosensitivity.

#### 5.4.2 Nanomedicine candidates and translational outlook

To date, no ferroptosis-immunotherapy nanocarrier has reached clinical testing. However, CNSI-Fe(II) represents a pioneering example of a nanomaterial-based ferroptosis inducer that has generated first-in-human data. Preclinical studies have shown that nanomedicine-based ferroptosis induction can overcome resistance to chemotherapy, radiotherapy, and targeted therapy. The first clinical trials for ferroptosis nanomedicines in lung cancer are expected within 2–3 years, initially targeting patients with therapy-resistant advanced non-small cell lung cancer.

#### 5.4.3 Key translational barriers

Several major barriers continue to hinder the clinical translation of ferroptosis-based therapies. First, first-generation ferroptosis inducers have limited drug-like properties, including poor solubility, rapid clearance, and off-target toxicity. Second, tumor-intrinsic heterogeneity creates diverse ferroptosis sensitivities, reinforcing the need for biomarker-guided patient selection rather than uniform therapeutic strategies. Third, the tumor microenvironment, including stromal buffering, hypoxia, acidosis, and immune cell vulnerability, profoundly modulates ferroptosis, making therapeutic induction inherently context-dependent. Fourth, the lack of validated pharmacodynamic biomarkers to confirm ferroptosis induction in patients remains a critical gap. Finally, the safety profile of systemic ferroptosis activation is incompletely understood; while CNSI-Fe(II) has shown acceptable local tolerability, concerns remain about off-target lipid peroxidation in normal organs. Addressing these barriers will require integrated biomarker development, optimized combination strategies, and next-generation ferroptosis inducers with improved tumor selectivity.

### 5.5 Strategic solutions and future directions for translational barriers

Identifying translational barriers is necessary, but charting pathways to overcome them is equally critical for advancing ferroptosis nanomedicine toward clinical reality. Below we propose targeted strategies to address each of the major barriers discussed in preceding sections.

#### 5.5.1 Addressing safety and off-target toxicity

To mitigate off-target ferroptosis in normal organs, future nanoplatforms should prioritize tumor-selective activation mechanisms (Hu and Loizou, 2007). Tumor-microenvironment (TME)-responsive designs, including pH-, GSH-, H_2_O_2_-, and enzyme-triggered release, remain the most promising strategy for localizing ferroptotic pressure to malignant tissues. Emerging approaches such as ultrasound-, light-, and X-ray-activated platforms can further confine ferroptosis induction to irradiated tumor regions. Additionally, iron-free ferroptosis inducers, such as FIN56 and FINO_2_, delivered via TME-responsive nanocarriers may reduce metal-associated long-term toxicity while preserving therapeutic efficacy. Long-term biodistribution studies using radiolabeled or mass-spectrometry-based imaging techniques should become a prerequisite for clinical candidate nomination.

#### 5.5.2 Overcoming manufacturing and pharmacokinetic heterogeneity

Batch-to-batch reproducibility of nanomedicines remains a formidable challenge that demands solutions beyond traditional good manufacturing practice (GMP) compliance. The integration of microfluidic manufacturing platforms has demonstrated superior control over particle size, polydispersity, and drug loading, enabling reproducible production of complex multi-component nanoplatforms (Webb et al., 2020). Machine learning and artificial intelligence-assisted formulation optimization can accelerate the identification of optimal synthesis parameters while reducing trial-and-error cycles (Dong et al., 2023). For regulatory harmonization, establishing standardized reference materials and consensus characterization protocols, including mandatory reporting of size, zeta potential, drug loading, release kinetics, and protein corona composition, would facilitate cross-study comparisons and regulatory review (Sharifi et al., 2022).

#### 5.5.3 Developing clinically validated ferroptosis biomarkers

Biomarker development must evolve from candidate discovery to clinical qualification (Luo et al., 2018). Multiplexed pharmacodynamic panels combining lipid peroxidation products (4-HNE, malondialdehyde, 8-iso-PGF_2_α), redox status markers (GSH/GSSG ratio, GPX4 activity), and iron handling parameters (ferritin, transferrin saturation, labile iron pool) may provide composite signatures of ferroptosis engagement. Emerging liquid biopsy approaches, including circulating tumor DNA methylation patterns and extracellular vesicle-associated ferroptosis markers, offer non-invasive, real-time monitoring of ferroptosis induction (Yang Y. C. et al., 2024). Prospective validation in clinical trials should incorporate these biomarkers as exploratory endpoints to establish thresholds for “ferroptosis response” and enable patient stratification. The establishment of a publicly accessible ferroptosis biomarker database would further accelerate clinical qualification.

#### 5.5.4 Rational patient selection and personalized strategies

Tumor-intrinsic heterogeneity in ferroptosis sensitivity necessitates biomarker-guided patient selection rather than universal application. Tumors with high GPX4 expression or low iron bioavailability may be resistant to iron-based ferroptosis inducers, while tumors with constitutively high lipid peroxidation or mitochondrial dysfunction may be especially vulnerable. Pre-treatment tumor biopsies should be molecularly profiled for ferroptosis-regulatory networks, including GPX4, SLC7A11, FSP1, DHODH, ACSL4, and iron metabolism genes (Zhou et al., 2024). Patients with “ferroptosis-high” signatures would be prioritized for ferroptosis-based regimens, while those with low signatures may benefit from combination strategies that prime ferroptosis vulnerability (e.g., radiotherapy- or chemotherapy-induced oxidative stress). Adaptive trial designs incorporating biomarker-based treatment allocation could accelerate ide ntification of responsive patient subsets (Ha et al., 2024).

#### 5.5.5 Enhancing combination therapy and immune synergy

The evolving understanding of ferroptosis-immunity crosstalk opens several future directions. First, selecting immunotherapy partners should be guided by the immune microenvironment, PD-1/PD-L1 or CTLA-4 checkpoint inhibitors may be optimal in immunologically “cold” tumors, whereas STING agonists or TLR agonists may be more suitable in “hot” tumors to avoid over-inflammation. Second, the timing of ferroptosis induction relative to immune checkpoint blockade requires optimization, as excessive ferroptosis may trigger immunosuppressive myeloid cell recruitment and TGF-β release (Sanavro et al., 2022). Third, the emerging field of “ferroptosis-epigenetics” suggests that epigenetic modulators could rewire ferroptosis sensitivity by altering lipid metabolism gene expression, offering new combination opportunities (Jin et al., 2025). Finally, the development of smart nanoplatforms that sequentially release ferroptosis inducers and immunomodulators in a programmed manner may maximize synergistic efficacy while minimizing toxicity.

#### 5.5.6 Next-generation nanoplatform design principles

Looking beyond incremental improvements, several transformative design principles are on the horizon. First, “all-in-one” nanoplatforms that integrate diagnosis (imaging), therapy (ferroptosis induction), and treatment monitoring (response tracking) — theranostic systems—should prioritize clinical interpretability over maximal complexity. Second, bioinspired and biomimetic designs may offer superior immune evasion, enhanced tumor penetration, and reduced immunogenicity compared to synthetic carriers. Third, biodegradable and renally clearable nanoplatforms, such as those based on organic frameworks, porous silicon, or polypeptide assemblies, could address long-term metal accumulation concerns without sacrificing therapeutic efficacy (Song et al., 2022). Fourth, CRISPR-based or RNA-interference (RNAi) approaches delivered via nanocarriers may enable transient suppression of ferroptosis defense pathways in a tumor-localized manner, circumventing the toxicity associated with systemic genetic ablation.

#### 5.5.7 Building a translational pipeline

Translational acceleration will ultimately depend on coordinated efforts across academia, industry, and regulatory agencies. Academic researchers should prioritize mechanistic rigor and reproducibility, reporting negative results as well as positive outcomes to inform the field. Industry partnerships should focus on scalable synthesis, formulation stability, and cost-effective production. Regulatory agencies should establish guidelines specifically for ferroptosis-inducing nanomedicines, including standardized toxicology panels, pharmacodynamic endpoints, and expedited pathways for first-in-class agents. Finally, the establishment of multi-center preclinical consortia, analogous to oncology cooperative groups, could harmonize *in vivo* efficacy testing across laboratories and accelerate candidate prioritization (Buchsbaum, 2025).

## 6 Conclusion

Ferroptosis-targeted cancer therapy has matured from a cell-death concept into a rich preclinical therapeutic field. Its biological rationale is compelling because cancer cells can depend on altered iron metabolism, lipid remodeling and antioxidant defenses, and because resistant tumor states may remain vulnerable to ferroptotic injury. Nanomedicine gives this biology a practical engineering language by controlling where ferroptosis pressure is delivered, how it is activated and which complementary therapies amplify it.

The strongest evidence supports several convergent design principles. Direct ferroptosis induction benefits from combining iron supply or Fenton-like catalysis with antioxidant-defense disruption. Precision delivery benefits from biomimetic membranes, exosomes, receptor targeting and TME-responsive release. Combination therapy is most persuasive when phototherapy, sonodynamic therapy, chemotherapy, radiotherapy or immunotherapy provides a mechanistic amplifier rather than an additive label. Immune synergy is especially promising, but ferroptosis-induced inflammation and suppressive myeloid responses must be tuned rather than assumed to be beneficial.

The main bottleneck is no longer whether ferroptosis can be induced in tumors. The central question is whether ferroptosis can be induced selectively, reproducibly and safely in patients. Addressing this question will require standardized pharmacokinetic reporting, long-term biodistribution studies, degradable and manufacturable materials, validated ferroptosis biomarkers, and patient-selection strategies grounded in tumor redox and immune state. The next phase of ferroptosis nanomedicine should therefore shift from increasingly complex proof-of-concept platforms to clinically interpretable systems that make the therapeutic mechanism, safety profile and target population clear. Strategically, the path forward lies in parallel development of TME-responsive activation mechanisms, artificial intelligence-accelerated manufacturing optimization, clinically qualified pharmacodynamic biomarkers, and adaptive trial designs that enable biomarker-guided patient allocation. Equally important are rigorous long-term biodistribution and degradation studies using standardized preclinical models, harmonized characterization protocols to facilitate regulatory review, and collaborative consortia that bridge academic discovery with industrial scalability. Ultimately, the success of ferroptosis nanomedicine will be measured not by the sophistication of nanoplatform designs, but by the clarity with which we can identify which patients benefit, monitor therapeutic engagement, and ensure durable clinical responses. The next phase of ferroptosis nanomedicine should therefore shift from increasingly complex proof-of-concept platforms to clinically interpretable systems that make the therapeutic mechanism, safety profile and target population clear.

## Glossary

ACSL4: Acyl-CoA synthetase long-chain family member 4
CD47: Cluster of differentiation 47
CDT: Chemodynamic therapy
cGAS-STING: Cyclic GMP-AMP synthase-stimulator of interferon genes
CRISPR: Clustered regularly interspaced short palindromic repeats
Cu: Copper
DHODH: Dihydroorotate dehydrogenase
EPR: Enhanced permeability and retention
FABP5: Fatty acid-binding protein 5
Fe: Iron
FSP1: Ferroptosis suppressor protein 1
GPX4: Glutathione peroxidase 4
GSH: Glutathione
HCC: Hepatocellular carcinoma
HMGB1: High-mobility group box 1
LMP: Lysosomal membrane permeabilization
LPCAT3: Lysophosphatidylcholine acyltransferase 3
MHC-I: Major histocompatibility complex class I
Mn: Manganese
MOF: Metal-organic framework
NIR-II: Near-infrared II
PDT: Photodynamic therapy
PROTAC: Proteolysis-targeting chimera
PSMA: Prostate-specific membrane antigen
PTT: Photothermal therapy
RFA: Radiofrequency ablation
ROS: Reactive oxygen species
RRM2: Ribonucleotide reductase regulatory subunit M2
SLC7A11: Solute carrier family 7 member 11
TME: Tumor microenvironment
